# Cinobufacini Injection Improves the Efficacy of Chemotherapy on Advanced Stage Gastric Cancer: A Systemic Review and Meta-Analysis

**DOI:** 10.1155/2018/7362340

**Published:** 2018-09-04

**Authors:** Xing Zhang, Yuan Yuan, Yupeng Xi, Xinyao Xu, Qiujun Guo, Honggang Zheng, Baojin Hua

**Affiliations:** ^1^Graduate School, Beijing University of Chinese Medicine, Beijing 100010, China; ^2^Department of Oncology, Guang'anmen Hospital, China Academy of Chinese Medical Sciences, Beijing 100010, China

## Abstract

Gastric cancer has a high morbidity and mortality. Chemotherapy regimens are routine advanced stage gastric cancer (AGC) treatment protocols, but most of these drugs have side-effects such as myelosuppression and gastrointestinal disorders. Cinobufacini, an extractive from TCM, could suppress cell proliferation and inhibit gastric cancer. In this study, we comprehensively reviewed the literature on the efficacy comparison between Cinobufacini injection combined with chemotherapy and chemotherapy solely used in AGC treatment. We extracted data for from six electronic databases to evaluate the efficacy of Cinobufacini injection on AGC patients. Twelve studies with a total of 853 patients were finally included in our study. The results indicated that Cinobufacini injection could increase response rate and disease control rate of chemotherapy on AGC, improve the life quality of AGC patients, increase leukocytes, improve anemia, improve hand-foot syndrome induced by chemotherapy, and relieve cancer pain. This study has its own limitations that prevented us from drawing a definite conclusion and more well-designed clinical trials of TCM are needed.

## 1. Introduction

Gastric cancer (GC) is one of the most common and lethal cancers worldwide and quite a number of GC patients are initially diagnosed with advanced stage gastric cancer (AGC) including local advanced GC (stage III and unresectable) and metastasis GC (stage IV). Chemotherapy regimens, such as FOLFOXs regimen (oxaliplatin, 5-fluorouracil, and leucovorin calcium), XELOX regimen (oxaliplatin and capecitabine), or other chemotherapeutic drugs, including paclitaxel, cisplatin, epirubicin, and etoposide [1, 2], are common AGC treatment protocols. But most of these drugs have side-effects such as myelosuppression (anemia, low count of leukocytes) and gastrointestinal tract disorders (nausea, vomiting, and diarrhea).

Traditional Chinese medicine (TCM) honors a long history in tumor treatment and it is accepted that TCM can inhibit tumor growth and metastasis, improve antitumor immunity, relieve tumor pains, and reduce side-effects of chemotherapy [3–5]. Combined treatment of TCM and modern medicine is widely used for AGC in China and studies showed TCM had an important potential value for improving the prognosis of patients with AGC [6, 7].

Cinobufacini (also called* Huachansu *in Chinese), extracted from the skins and parotid venom glands of the* Bufo bufo gargarizans Canto*, is a kind of traditional Chinese animal-derived drug used in the treatment of malignant neoplasms in ancient oriental countries. Recent studies showed that Cinobufacini could induce the apoptosis of tumor cells and downregulate protumor inflammatory signaling pathways in the tumor microenvironment [8–11]. Furthermore, researches also indicated that Cinobufacini can inhibit several kinds of human tumors in both clinical treatments and animal xenograft models [12–14].

While Cinobufacini antitumor activity has been proved, the gastrointestinal metabolic pathways of Cinobufacini remain unclear, so intravenous administration (e.g., Cinobufacini injection) is the most common route. Thus, Cinobufacini injection was increasingly used in clinical and basic studies. As there is no systemic review specifically for Cinobufacini injection on AGC treatment, this systematic review and meta-analysis comprehensively evaluated the effects of it according to the PRISMA statement for a high quality [15, 16].

## 2. Material and Methods

### 2.1. Literature Search

Studies were explored from databases including PubMed (from Jan. 1975 to Oct. 2017), Cochrane library (from Jan. 2010 to Oct. 2017), Excerpta Medica data BASE (Embase) (from Jan. 1990 to Oct. 2017), China National Knowledge Infrastructure (CNKI) (from Jan. 1979 to Oct. 2017), Weipu database (VIP) (from Jan. 1990 to Oct. 2017), and Wanfang database (WF) (from Jan. 1989 to Oct. 2017). All the studies were searched regardless of their publication types and without language restriction. The search terms were as follows: “Cinobufacini” OR “Cinobufotalin” OR “Huachansu” AND “gastric” OR “stomach”. In addition to electronic databases, printed journals and relevant textbooks were manually searched from the libraries of Beijing University of Chinese Medicine, Peking Union Medical College and Guang'anmen Hospital. Specialized experts in particular fields were consulted for necessary supplements as well.

Inclusion criteria include the following: (1) types of studies: randomized clinical trials (RCTs); (2) participants: adult human populations (*⩾*18 years old) who were pathologically diagnosed as gastric cancer with clinical stages III (unresectable) and IV; (3) interventions: the control group was treated with chemotherapy while the experimental group was treated with the same chemotherapeutics plus Cinobufacini injection; and (4) outcomes: short/long-term chemotherapy response rate, Karnofsky's performance score, chemotherapeutic side-effects such as myelosuppression and gastrointestinal symptoms, and pain management. Exclusion criteria include the following: (1) studies such as reviews, animal researches, observational studies without control group, or other kinds of non-RCT studies; (2) trails about other types of gastrointestinal diseases; (3) participants who had nonpathological diagnosis, previously subjected to chemotherapy, radiotherapy or surgery, concurrent infection, or other malignancies or severe illnesses; and (4) participants in the control group who were treated with other antitumor TCM drugs.

### 2.2. Literature Selection and Data Extraction

Two independent reviewers (Yuan Y, Qiujun G) evaluated each title, abstract, citation, and selected relevant studies according to the inclusion criteria. Disagreements were discussed with and resolved by the third reviewer (Zizhen Y). Data from included studies were extracted separately by Yupeng X by using a specific form and checked by Xing Z. The characteristics of the data included name of first author, year of publication, gender and number of cases and controls, methods of randomization, interventions, treatment period, and outcomes. The hazard ratio (HR) was calculated from the Kaplan-Meier survival curve and survival outcome events as reported by Tierney [29].

### 2.3. Quality Assessment of Studies

The methodological quality of each randomized controlled trials (RCTs) was independently assessed by Yuan Y and Qiujun G via the Cochrane Risk of Bi as tool [30]. Disagreements were discussed with and resolved by Baojin H.

### 2.4. Data Synthesis and Analyses

The statistical analyses were performed using Review Manager (RevMan) 5.3.5 software (Cochrane Community, London, United Kingdom) and STATA 14 software. The total effectiveness rates of dichotomous data were pooled using risk ratios (RRs) with 95% confidence interval (CI). P < 0.05 was considered to indicate a statistically significant difference. The heterogeneity of the included studies was evaluated by the *χ*^2^ and I^2^ tests, and P < 0.10 or I^2^ > 50% was defined as indicating heterogeneity. The fixed-effect models were used in merging homogeneity data and the random-effects models were applied to merge of heterogeneous data. The publication bias was evaluated by visual assessment of the asymmetry of funnel plots (RevMan 5.3.5) and Egger's test (STATA 14) with p < 0.05 indicating potential bias. The sensitive analysis was evaluated by reanalyzing the data using different statistical approaches or eliminating a variable which takes the largest proportion.

## 3. Results

### 3.1. Included Eligible Studies

**Figure 1:**
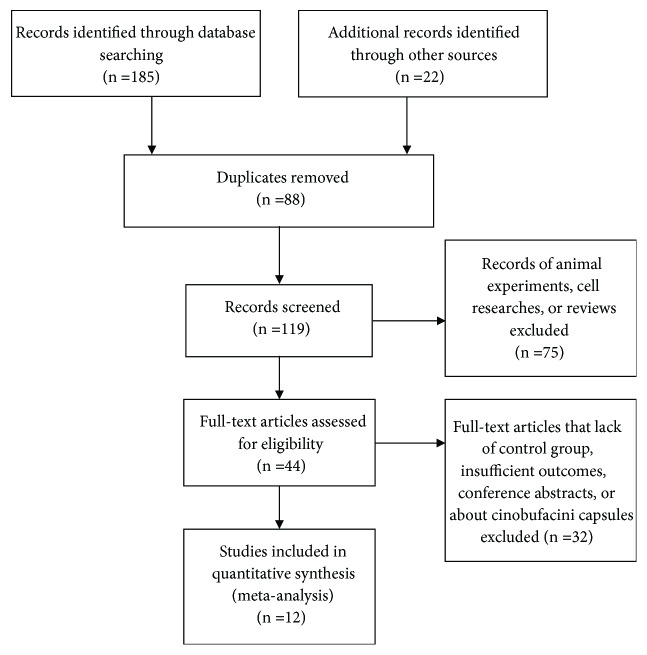
Flow diagram of the literature search process.

207 studies (including 22 additional records identified through other sources such as postgraduate dissertations and conference articles) were initially searched out by using the search strategy mentioned above, among which 88 duplicated studies were removed, and 75 studies were excluded because they were animal experiments, cell researches, or reviews. After reading the full text, 32 studies were excluded because they lacked control group, had insufficient outcomes conference abstracts, or were about Cinobufacini capsules. Eventually, 12 studies were included in the final research (Figure 1).

#### 3.1.1. Characteristics of Included Studies

Twelve studies with a total of 853 patients were finally included (423 patients in the experiment group and 430 patients in the control group). Characteristics such as sample size, gender, age, interventions, and outcomes of each study were described in Table 1.

#### 3.1.2. Quality Assessment of Included Studies

**Figure 2:**
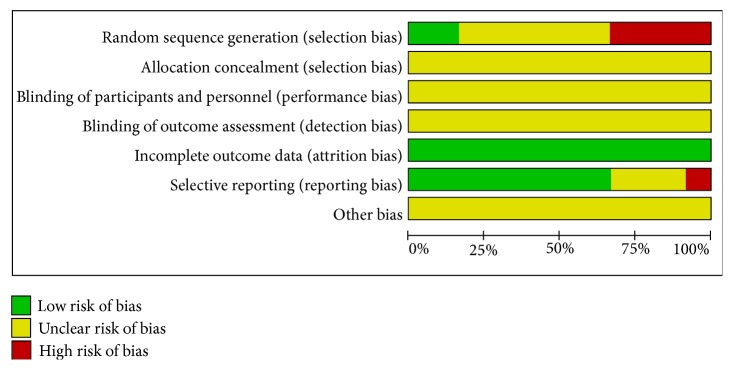
Risk of bias graph.

**Figure 3:**
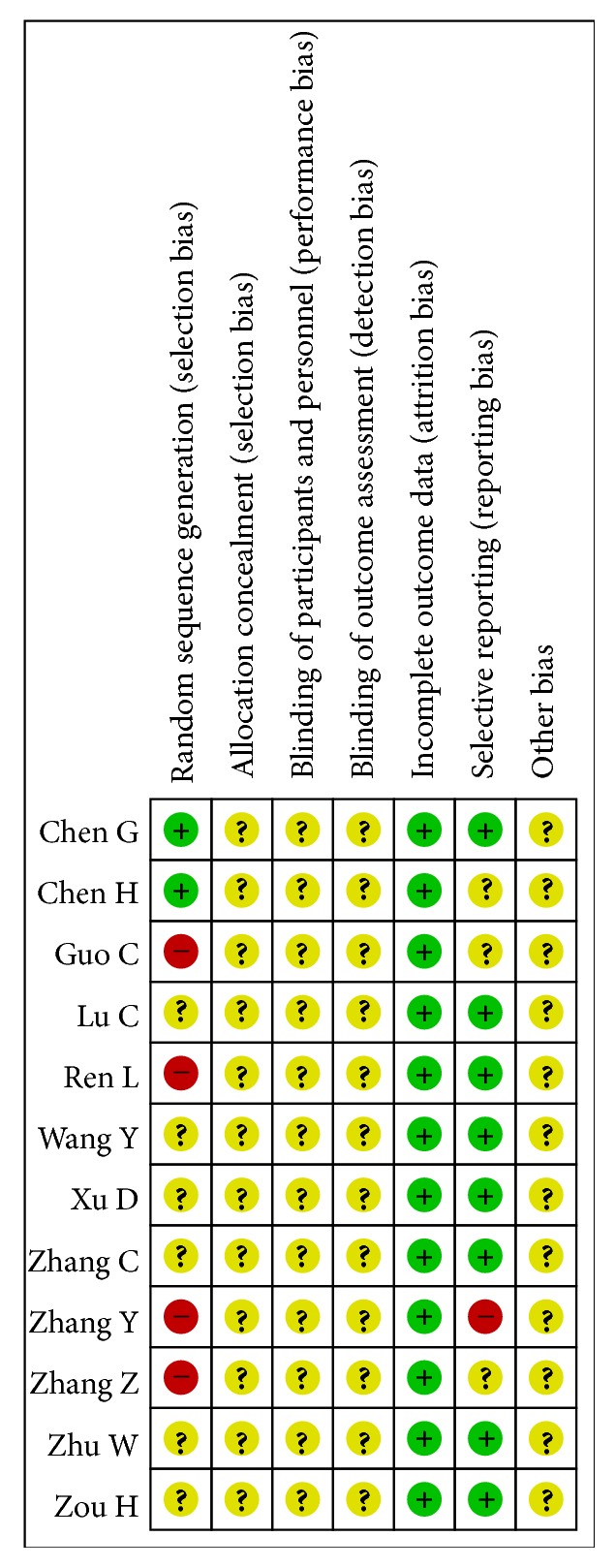
Risk of bias summary.

All of the included studies applied randomization methodology, but six of them did not describe the detailed random method. All of the included studies had complete data but none of them mentioned the details of allocation concealment and blinding of participants and personnel and outcome assessment. One study had high risk of reporting bias for its incompleteness of outcome, so it cannot be entered in the meta-analysis (Figures 2 and 3).

### 3.2. Meta-Analysis of Cinobufacini Injection on AGC Treatment

**Figure 4:**
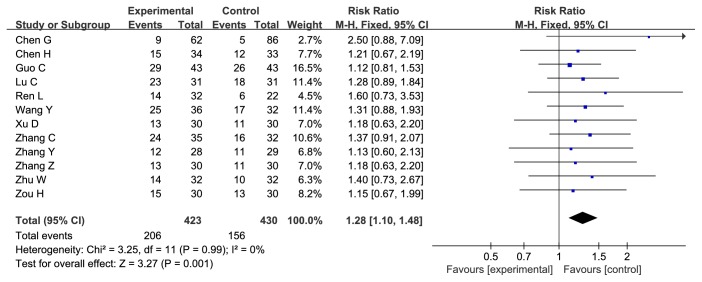
**Forest plot of RR (risk ratio) for evaluation of response rate in fixed-effect model.** The RR of chemotherapy response rate in Cinobufacini injection and chemotherapy group was compared with the chemotherapy group. Individual study is shown in the square with blue color, and the pooled datasets were shown in the diamond, representing the 95% confidence interval (CI) of each study. RR > 1 implied a better chemotherapy response rate of the experimental group. The size of each investigation represented the weighting factor (1/SE) assigned to the study.

*Cinobufacini Injection Could Enhance Response Rate (RR) of Chemotherapy on AGC*. All of the twelve studies evaluated the RR. The RR in the experiment group (Cinobufacini injection combined with chemotherapy) was significantly higher than that in the control group (chemotherapy only), with the risk ratio = 1.28, 95% CI: 1.10-1.48, P = 0.001 in the Z test. The result did not indicate the heterogeneity with the Chi^2^ = 3.25, df = 11, P = 0.99, I^2^ = 0% (Figure 4).

**Figure 5:**
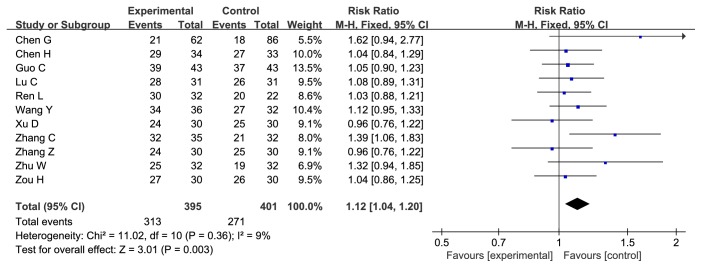
**Forest plot of RR for evaluation of disease control rate in fixed-effect model.** The RR of disease control rate in Cinobufacini injection and chemotherapy group was compared with the chemotherapy group. Individual study is shown in the square with blue color, and the pooled datasets were shown in the diamond, representing the 95% confidence interval (CI) of each study. RR > 1 implied a better disease control rate of the experimental group. The size of each investigation represented the weighting factor (1/SE) assigned to the study.

*Cinobufacini Injection Could Enhance Disease Control Rate (DCR) of Chemotherapy on AGC*. Eleven studies evaluated the DCR which in the experiment group was significantly higher than that in the control group, with the risk ratio = 1.12, 95% CI: 1.04-1.20, P = 0.003 in the Z test. The result did not indicate the heterogeneity with the Chi^2^ = 11.02, df = 10, P = 0.36, I^2^ = 9% (Figure 5).

**Figure 6:**
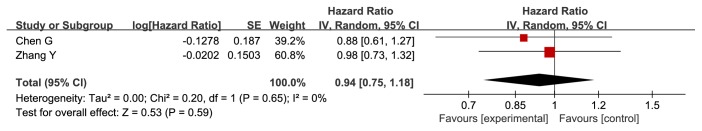
**Forest plot of HR (hazard ratio) for evaluation of overall survival in fixed-effect model.** The HR of overall survival in Cinobufacini injection and chemotherapy group was compared with the chemotherapy group. Individual studies are shown in the red-colored squares, and the pooled datasets are shown by the diamond, representing the 95% confidence interval (CI) of each study. HR < 1 implied improved overall survival in the experimental group. The size of each investigation represented the weighting factor (1/SE) assigned to the study.

*Cinobufacini Injection Could Not Prolong the Overall Survival Time (OS) of AGC Patients. *Two studies evaluated the OS of AGC patients. We pooled the hazard ratios (HRs) of OS and the result showed that pooled HR = 0.94, with 95% CI: 0.75-1.18, P = 0.59 in the Z test. The result did not indicate the heterogeneity with the Chi^2^ = 0.20, df = 1, P = 0.65, I^2^ = 0% (Figure 6).

*Cinobufacini Injection Could Improve the Life Quality of AGC Patients.* KPS is a recognized method for evaluating the quality of life, scoring integer 100 to 0 degressively with the decreased quality of life. Six studies included the KPS evaluation. Cinobufacini injection could improve KPS (KPS enhancement ≥ 10) when combined with chemotherapy, with the risk ratio = 1.83, 95% CI: 1.40-2.39, P < 0.00001 in the Z test. The result did not indicate the heterogeneity with the Chi^2^ = 4.61, df = 5, P = 0.46, I^2^ = 0% (Table 2).

*Cinobufacini Injection Could Reduce the Declination of Leucocyte Count but Could Not Inhibit the Severe Declination (III-IV Degrees)*. Six studies evaluated the low count of leukocytes of AGC patients. As the result showed Chi^2^ = 10.08, df = 5, P = 0.07, I^2^ = 50% which indicated possible heterogeneity. The P values of Z test between experimental group and control group were 0.04 (random-effect model). These results indicated Cinobufacini injection could improve the situation of the low count of leukocytes due to the chemotherapy (Table 2). Four studies evaluated the severe situation of low count of leukocytes and the results showed that Cinobufacini injection could not inhibit the III-IV-degree declination of leukocytes count, with the risk ratio = 0.61, 95% CI: 0.33-1.14, P = 0.12 in the Z test. The result did not indicate the heterogeneity with the Chi^2^ = 3.77, df = 3, P = 0.29, I^2^ = 20% (Sup 2, Fig 3).

*Cinobufacini Injection Could Reduce the Morbidity of (Severe) Nausea and Vomiting Caused by Chemotherapy.* Five studies evaluated the incidence of nausea and vomiting between the two groups and the results showed a significant difference with the risk ratio = 0.68, 95% CI: 0.53-0.86, P = 0.001 in the Z test. The results did not indicate the heterogeneity with the Chi^2^ = 7.52, df = 4, P = 0.11, I^2^ = 47% (Table 2). The similar results were seen in four studies that involved Grades III-IV of nausea and vomiting, with the risk ratio = 0.34, 95% CI: 0.14-0.82, P = 0.02 in the Z test. The results did not indicate the heterogeneity with the Chi^2^ = 3.11, df = 3, P = 0.37, I^2^ = 4% (Sup 2, Fig 5).

*Cinobufacini Injection Could Alleviate Hand-Foot Syndrome (HFS) Induced by Chemotherapy*. Some chemotherapeutic drugs such as novel-fluorouracil derivatives could induce HFS sluggish feelings and red or black spots on hands and feet. Three studies evaluated number of HFS cases and the results showed Cinobufacini injection could reduce the morbidity of HFS. The result showed a significant difference with the risk ratio = 0.55, 95% CI: 0.33-0.91, P = 0.02 in the Z test. The results did not indicate heterogeneity with the Chi^2^ = 1.48, df = 2, P = 0.48, I^2^ = 0% (Table 2).

*Cinobufacini Injection Could Relieve Tumor Pain.* Two studies were conducted to evaluate the effectiveness of Cinobufacini injection in managing cancer pain. The result indicated that Cinobufacini injection significantly relieves pain with the risk ratio = 0.1.81, 95% CI: 1.30-2.54, P = 0.0.0005 in the Z test. The result did not indicate heterogeneity with the Chi^2^ = 0.12, df = 1, P = 0.73, I^2^ = 0% (Table 2).

Cinobufacini injection could not reduce the incidence of anemia, diarrhea, peripheral neurotoxicity, and oral mucositis caused by chemotherapy (Sup 2, Fig 8-11).

Three studies were conducted to compare the incidence of anemia between experimental and control groups. There were no significant differences in the incidence of anemia between two groups, with the risk ratio = 0.79, 95% CI: 0.58-1.08, P = 0.14 in the Z test. The results did not indicate heterogeneity with the Chi^2^ = 0.37, df = 2, P = 0.83, I^2^ = 0%.

Cinobufacini injection could not reduce the morbidity of diarrhea induced by chemotherapy. There was no significant difference between the two groups, with the risk ratio = 0.77, 95% CI: 0.52-1.15, P = 0.21 in the Z test. The results did not indicate the heterogeneity with the Chi^2^ = 1.65, df = 4, P = 0.80, I^2^ = 0%. The similar results were shown in four studies that involved the incidence of III-IV degree diarrhea with the risk ratio = 0.33, 95% CI: 0.08-1.38, P = 0.13 in the Z test. The results did not indicate heterogeneity with the Chi^2^ = 0.18, df = 2, P = 0.91, I^2^ = 0%.

Cinobufacini injection could not reduce the incidence of peripheral neurotoxicity and oral mucositis. Three studies and two studies evaluated the recurrence of peripheral neurotoxicity and oral mucositis accordingly. There were no significant differences between experimental group and control group in the incidence of peripheral neurotoxicity and oral mucositis with the P = 0.23 and 0.39 accordingly. Significant heterogeneities were detected with P < 0.0.01 and I^2^ = 91% and 88% accordingly.

### 3.3. Sensitivity Analysis

We conducted the sensitivity analysis to strengthen the reliability of the results of response rate and disease control rate. The sensitivity analysis showed the same effect sizes among a fixed-effect model and a random-effect model of the response rate analysis, disease control rate analysis, and hazard ratio analysis. The same effects were shown in other outcome measures in the sensitive analysis except in the analysis of Grades III-IV nausea and vomiting, peripheral neuropathy, and oral mucositis (Table 2). By taking into consideration the heterogeneity, we adopted the corresponding result when there were inconsistent results in sensitivity analysis.

### 3.4. Publication Bias

**Figure 7:**
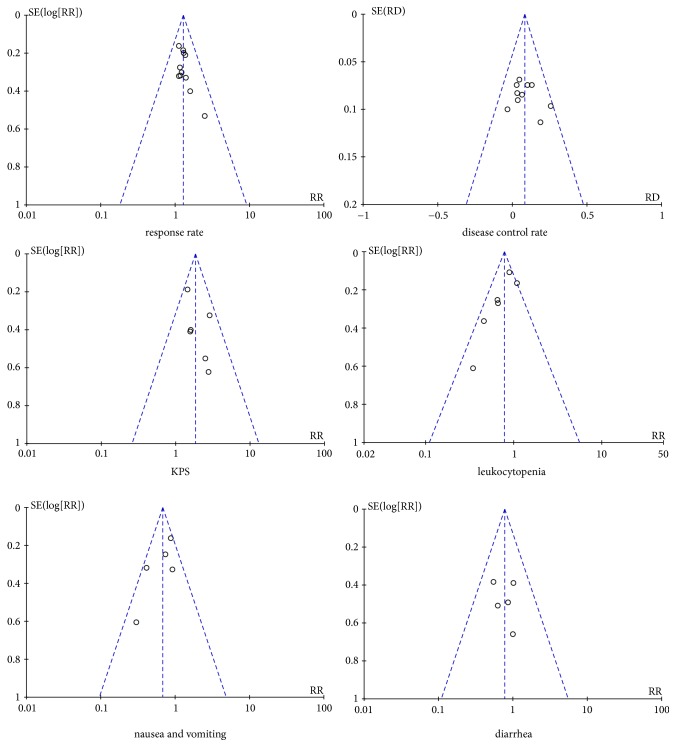
Funnel plots of response rate, disease control rate, KPS, leukocytopenia, and nausea, vomiting, and diarrhea.

The funnel plots (Figure 7) were not strictly symmetrical in the meta-analysis of response rate, disease control rate, KPS, and diarrhea. But Egger's test (Table 3) showed that there was no significant publication bias among the studies except the meta-analysis of disease control rate (P = 0.004) and diarrhea (P = 0.026).

## 4. Discussion

Gastric cancer has a high morbidity around the world. The comprehensive treatment including surgery, chemotherapy, radiotherapy, targeted therapy, support treatment, and treatment of TCM is the optimal treatment for gastric tumor. Chemotherapy is one of the most important treatments for advanced gastric cancer (AGC), but the response rate is far from satisfactory so far. The combination of TCM and modern medical treatments has been proved effective on AGC. For instance, a research showed that TCM herbal formula of invigorating spleen could prolong the median overall survival time and improve the prognosis of patients with AGC [7]. On fundamental research,* A. cucullata*, an extractive from TCM herb* Alocasia cucullata (Lour.) G. Don*, was reported to have a potent antigastric cancer activity both in vitro and in vivo via antiproliferation of G0/G1 arrest and cell proapoptosis, including PI3K/Akt pathway, ERK activity, stimulated cytochrome C release, and caspase 3/7 activity accompanied with an increase of Bax/Bcl-2 ratio [31]. As a kind of TCM extractive, Cinobufacini could suppress the cell proliferation of BGC-823 human gastric cancer cells via targeting BAG-1 (an antiapoptosis gene) and inhibit tumor growth and metastasis in xenograft models [8, 14, 32]. These may partially explain the mechanisms of how TCM and Cinobufacini injection inhibit gastric cancer. Some researchers started to work on the antitumor components of Cinobufacin injection, and Bufadienolides might be one of the antitumor agents in treating gastric cancer [33]. Further studies are needed to clarify how Cinobufacini injection could benefit cancer patients.

In this review, we comprehensively reviewed the literature on the efficacy comparison between Cinobufacini injection combined with chemotherapy and chemotherapy solely used in AGC treatment. Our results indicated that Cinobufacini injection could enhance the response rate and disease control rate of chemotherapy, which meant the experiment group had a better short-term efficacy than that in the control group. However, due to insufficient data, only two of our included studies included overall survival time, and our results showed that Cinobufacini injection could not prolong the overall survival time. High life quality is also important for tumor patients' living and recovery. Our study showed that Cinobufacini injection improved the life quality of AGC patients receiving chemotherapy by enhancing their KPS.

Side-effects such as myelosuppression and gastrointestinal toxicity constantly occur in tumor patients undergoing chemotherapy, which cause them great trouble. TCM plays an important role in alleviating side-effects when used in combination with chemotherapy. For instance, a double-blind clinical trial showed that the standardized ginger extract (the extract from a kind of traditional medicine in Asian countries to treat nausea and vomiting) acted as an antiemetic against chemotherapy-induced nausea and vomiting [34]. A meta-analysis based on eight trails indicated that Chinese herb medicine significantly protected peripheral blood WBCs from decreasing during the course of chemotherapy or radiotherapy [35]. Astragalus membranaceus was also proved to have a myelo-protective and myelo-therapeutic capacity against the chemotherapy-induced myelosuppression, evidenced at both laboratory and morphological levels in basic study [36]. Our results indicated that Cinobufacini injection could inhibit the declination of leukocytes in peripheral blood and allay nausea and vomiting caused by chemotherapy, but it could not prevent myelosuppression or gastrointestinal toxicity which commonly present as anemia or diarrhea. Most tumor patients suffer from cancer pain, which even painkillers cannot cure. Some Chinese herbal injectionsare proved to improve clinical efficacy and relieve adverse reactions when combined with the FOLFOX regimen in treating gastric cancer [37]. Chinese medicine such as Fufang Kushen injection could reduce cancer pain directly by blocking TRPV1 signaling pathway [38]. Cinobufacini injection could help to relieve cancer pain as well based on our evaluation, but the exact mechanisms of these effects remain unclear.

However, this study has its own limitations. First, allocation concealment and blinding of all the included studies were unclear and there was publication bias in some evaluations since the included studies were all published in Chinese. Second, we failed to evaluate the long-term effects, because the treatment periods of included studies were generally short and they did not include long-term follow-ups. Thus, the long-term effects of Cinobufacini injection on AGC patients remain unclear. Third, the criteria for the evaluation of tumor response varied from one study to another, which might bring different results in subgroup analysis in RR and DCR evaluation. Taking into consideration all the above reasons, the evidence for this study might be insufficient. Although the above questions might exist that prevent us from drawing a definite conclusion about Cinobufacini injection, our study still provided helpful information for clinical practice that Cinobufacini injection could enhance the efficacy of other treatments in AGC patients, reduce the side-effects induced by chemotherapy, and help to relieve cancer pain, which might be helpful for clinical medication. However, in order to draw precise conclusion, more well-designed clinical trials with long-term follow-ups of Cinobufacini injection are needed for future study.

## Figures and Tables

**Table 1:** Characteristics of the included studies.

Trials	Sample size (E/C)	Gender	Age (yr)	clinical stage	Experimental group (E)	Control group (C)	Period	Outcome measure
Zhu W [17]	32/32	M: 16, F: 16/M: 15, F: 17	32-74 (61.7)/34-72 (62.8)	III: 15, IV: 17/III: 16, IV: 16	Cinobufacini injection 30 ml iv. Qd + C	Xelox regimen	4 weeks	tumor response (WHO), Kamofsky Score, Side-effects of chemotherapy (WHO)

Zou H [18]	30/30	M: 13, F: 17/M: 21, F: 9	59.1/56.5	III, IV	Cinobufacini injection 20 ml iv. Qd + C	EOF regimen	6 weeks	tumor response (RECIST), Kamofsky Score, Side-effects of chemotherapy (WHO)

Zhang C [19]	35/32	M: 28, F: 7/M: 23, F: 9	46-82 (64)/42-79 (66)	III: 15, IV: 20/III: 13, IV: 19	Cinobufacini injection 20ml iv. Qd + C	ELF regimen	8 weeks	tumor response (UICC), Side-effects of chemotherapy (WHO)

Guo C [20]	43/43	M: 62, F: 24	43-74 (55)	IV	Cinobufacini injection 20 ml iv. Qd + C	Docetaxel	9 weeks	tumor response (WHO), Kamofsky Score, Side-effects of chemotherapy (WHO), analgesic effect

Zhang Y [21]	28/29	None	42-71 (57)/35-69 (54)	IV	Cinobufacini injection 50 ml iv. Qd + C	oxaliplatin + floxuridine	9 weeks	tumor response (WHO), Kamofsky Score, Side-effects of chemotherapy (WHO), analgesic effect, 1 year and 2 year survival time

Chen G [22]	62/86	M: 56, F: 30/M: 39, F: 23	65-87 (71.8 ± 18.6)/64-89 (73.1 ± 22.3)	IV	Cinobufacini injection 10 ml iv. Tid + C	Capecitabine	6 weeks	tumor response (WHO), Kamofsky Score, Side-effects of chemotherapy (WHO), overall survival time

Xu D [23]	30/30	M: 20, F: 10/M: 21, F: 9	66.3 ± 4.6/65.0 ± 3.9	IV	Cinobufacini injection 20 ml iv. Qd + C	Capecitabine	6 weeks	tumor response (WHO), Kamofsky Score, Side-effects of chemotherapy (WHO), analgesic effect

Zhang Z [24]	30/30	None	35-79 (53)/33-75 (56)	IV	Cinobufacini injection 20ml iv. Qd + C	Hydroxycamptothecin	6 weeks	tumor response (WHO), Kamofsky Score, Side-effects of chemotherapy (WHO), analgesic effect

Lu C [25]	31/31	M: 34 F: 28	37-71 (54 ± 17)	III	Cinobufacini injection 20 ml iv. Qd + C	FOLFOX4 regimen	9 weeks	tumor response (WHO), immune regulation

Wang Y [26]	36/32	M: 48 F: 20	40-72 (54)	IV	Cinobufacini injection 20 ml iv. Qd + C	FOLFOX4 regimen	8 weeks	tumor response (WHO), Side-effects of chemotherapy (WHO),

Ren L [27]	32/22	Unclear	40-68 (53)	IV	Cinobufacini injection 20 ml iv. Qd + C	FOLFOX regimen	6 weeks	tumor response (WHO), Side-effects of chemotherapy (WHO), Kamofsky Score,

Chen H [28]	34/33	M: 20, F: 14/M:20, F: 13	50.6/40.9	III: 23, IV: 11/III: 24, IV: 9	Cinobufacini injection 30 ml iv. Qd + C	TPF regimen	6 weeks	tumor response (WHO), Side-effects of chemotherapy (WHO)

ELF regimen: oxaliplatin + epirubicin + floxuridine; ELF regimen: etoposide + cisplatin+ floxuridine; Xelox regimen: oxaliplatin + capecitabine; FOLFOX4 regimen: oxaliplatin + floxuridine + leucovorin; and TPF regimen: paclitaxel + cisplatin +floxuridine.

**Table 2:** Meta-analysis of KPS, side-effects and tumor-related pain.

Meta-analysis	No. of study	Risk Ratio [%95 CI]				heterogeneity	
		Fix-model	P value	Random-model	P value	I^2^(%)	P value
KPS	6	1.83 [1.40, 2.39]	P < 0.00001	1.76 [1.35, 2.29]	P < 0.0001	0	0.46
leukocytopenia	6	0.78 [0.65, 0.93]	P = 0.007	0.76 [0.58, 0.99]	P = 0.04	50	0.07
Grades III-IV leukocytopenia	4	0.61 [0.33, 1.14]	P = 0.12	0.58 [0.23, 1.46]	P = 0.25	20	0.29
nausea and vomiting	5	0.68 [0.53, 0.86]	P = 0.001	0.68 [0.48, 0.96]	P = 0.03	47	0.11
Grades III-IV nausea and vomiting	4	0.34 [0.14, 0.82]	P = 0.02	0.44 [0.17, 1.13]	P = 0.09	4	0.37
hand-foot syndrome	3	0.55 [0.33, 0.91]	P = 0.02	0.54 [0.32, 0.91]	P = 0.02	0	0.48
tumor-related pain	2	1.81 [1.30, 2.54]	P = 0.0005	1.83 [1.31, 2.55]	P = 0.0004	0	0.73
anemia	3	0.79 [0.58, 1.08]	P = 0.14	0.80 [0.59, 1.09]	P = 0.15	0	0.83
diarrhea	5	0.77 [0.52, 1.15]	P = 0.21	0.76 [0.51, 1.14]	P = 0.19	0	0.8
peripheral neurotoxicity	3	0.64 [0.52, 0.80]	P < 0.0001	0.57 [0.23, 1.43]	P = 0.23	91	<0.00001
oral mucositis	2	0.46 [0.25, 0.83]	P = 0.01	0.37 [0.04, 3.47]	P = 0.39	88	0.004

**Table 3:** Egger's test.

Meta-analysis of publication bias	P value
response rate	0.114
disease control rate	0.004
KPS	0.250
leukocytopenia	0.224
nausea and vomiting	0.177
diarrhea	0.026

## Data Availability

All the data are included in this article and its supplementary information files.

## Supplementary Materials

Supplementary Materials Supplementary File: forest plots of KPS, side-effects, and tumor-related pain (DOC). Supplement 1: forest plots of response rate, overall survival, and disease control rate (DOC). Supplement 2: sensitivity analysis (DOC). Supplement 3: publication bias of the meta-analysis (DOC).
