# Supplementary material for: Cinobufacini Injection Improves the Efficacy of Chemotherapy on Advanced Stage Gastric Cancer: A Systemic Review and Meta-Analysis
Source: Evid Based Complement Alternat Med. 2018 Sep 4;2018:7362340. doi: 10.1155/2018/7362340 (PMC6142757; doi:10.1155/2018/7362340)
Supplement: Supplementary Materials — Supplementary File: forest plots of KPS, side-effects, and tumor-related pain (DOC). Supplement 1: forest plots of response rate, overall survival, and disease control rate (DOC). Supplement 2: sensitivity analysis (DOC). Supplement 3: publication bias of the meta-analysis (DOC). [file 7362340.f1.zip › Supplementary Materials/supplement.doc]

**Fig1. Forest plot of RR for evaluation of KPS in random effect model.** The RR of KPS in Cinobufacini injection and chemotherapy group was compared with the chemotherapy group. Individual study is shown in the square with blue color, and the pooled datasets were shown in the diamond, representing the 95% conﬁdence interval (CI) of each study. RR > 1 implied a higher KPS score of the experimental group. The size of each investigation represented the weighting factor (1/SE) assigned to the study.

**Fig2. Forest plot of RR for evaluation of leukocytopenia in fixed-effect model.** The RR of leukocytopenia in Cinobufacini injection and chemotherapy group was compared with the chemotherapy group. Individual study is shown in the square with blue color, and the pooled datasets were shown in the diamond, representing the 95% conﬁdence interval (CI) of each study. RR < 1 implied a reduced incidence of leukocytopenia of the experimental group. The size of each investigation represented the weighting factor (1/SE) assigned to the study.

**Fig3. Forest plot of RR for evaluation of Grade III-IV leukocytopenia in random effect model.** The RR of Grade III-IV leukocytopenia in Cinobufacini injection and chemotherapy group was compared with the chemotherapy group. Individual study is shown in the square with blue color, and the pooled datasets were shown in the diamond, representing the 95% conﬁdence interval (CI) of each study. RR < 1 implied a reduced incidence of Grade III-IV leukocytopenia of the experimental group. The size of each investigation represented the weighting factor (1/SE) assigned to the study.

**Fig4. Forest plot of RR for evaluation of anemia in random effect model.** The RR of anemia in Cinobufacini injection and chemotherapy group was compared with the chemotherapy group. Individual study is shown in the square with blue color, and the pooled datasets were shown in the diamond, representing the 95% conﬁdence interval (CI) of each study. RR < 1 implied a reduced incidence of anemia of the experimental group. The size of each investigation represented the weighting factor (1/SE) assigned to the study.

**Fig5. Forest plot of RR for evaluation of nausea and vomiting in random effect model.** The RR of nausea and vomiting in Cinobufacini injection and chemotherapy group was compared with the chemotherapy group. Individual study is shown in the square with blue color, and the pooled datasets were shown in the diamond, representing the 95% conﬁdence interval (CI) of each study. RR < 1 implied reduced incidence of nausea and vomiting of the experimental group. The size of each investigation represented the weighting factor (1/SE) assigned to the study.

**Fig6. Forest plot of RR for evaluation of Grade III-IV nausea and vomiting in random effect model.** The RR of Grade III-IV nausea and vomiting in Cinobufacini injection and chemotherapy group was compared with the chemotherapy group. Individual study is shown in the square with blue color, and the pooled datasets were shown in the diamond, representing the 95% conﬁdence interval (CI) of each study. RR < 1 implied reduced incidence of Grade III-IV nausea and vomiting of the experimental group. The size of each investigation represented the weighting factor (1/SE) assigned to the study.

**Fig7. Forest plot of RR for evaluation of diarrhea in random effect model.** The RR of diarrhea in Cinobufacini injection and chemotherapy group was compared with the chemotherapy group. Individual study is shown in the square with blue color, and the pooled datasets were shown in the diamond, representing the 95% conﬁdence interval (CI) of each study. RR < 1 implied reduced incidence of diarrhea of the experimental group. The size of each investigation represented the weighting factor (1/SE) assigned to the study.

**Fig8. Forest plot of RR for evaluation of Grade III-IV diarrhea in random effect model.** The RR of Grade III-IV diarrhea in Cinobufacini injection and chemotherapy group was compared with the chemotherapy group. Individual study is shown in the square with blue color, and the pooled datasets were shown in the diamond, representing the 95% conﬁdence interval (CI) of each study. RR < 1 implied reduced incidence of Grade III-IV diarrhea of the experimental group. The size of each investigation represented the weighting factor (1/SE) assigned to the study.

**Fig9. Forest plot of RR for evaluation of gastrointestinal tract disorders in fix effect model.** The RR of gastrointestinal tract disorders in Cinobufacini injection and chemotherapy group was compared with the chemotherapy group. Individual study is shown in the square with blue color, and the pooled datasets were shown in the diamond, representing the 95% conﬁdence interval (CI) of each study. RR < 1 implied reduced incidence of gastrointestinal tract disorders of the experimental group. The size of each investigation represented the weighting factor (1/SE) assigned to the study.

**Fig10. Forest plot of RR for evaluation of HFS in random effect model.** The RR of HFS in Cinobufacini injection and chemotherapy group was compared with the chemotherapy group. Individual study is shown in the square with blue color, and the pooled datasets were shown in the diamond, representing the 95% conﬁdence interval (CI) of each study. RR < 1 implied reduced incidence of HFS of the experimental group. The size of each investigation represented the weighting factor (1/SE) assigned to the study.

**Fig11. Forest plot of RR for evaluation of peripheral neurotoxicity in fix effect model.** The RR of peripheral neurotoxicity in Cinobufacini injection and chemotherapy group was compared with the chemotherapy group. Individual study is shown in the square with blue color, and the pooled datasets were shown in the diamond, representing the 95% conﬁdence interval (CI) of each study. RR < 1 implied reduced incidence of peripheral neurotoxicity of the experimental group. The size of each investigation represented the weighting factor (1/SE) assigned to the study.

**Fig12. Forest plot of RR for evaluation of oral mucositis in fix effect model.** The RR of oral mucositis in Cinobufacini injection and chemotherapy group was compared with the chemotherapy group. Individual study is shown in the square with blue color, and the pooled datasets were shown in the diamond, representing the 95% conﬁdence interval (CI) of each study. RR < 1 implied reduced incidence of oral mucositis of the experimental group. The size of each investigation represented the weighting factor (1/SE) assigned to the study.
