# Supplementary material for: Cinobufacini Injection Improves the Efficacy of Chemotherapy on Advanced Stage Gastric Cancer: A Systemic Review and Meta-Analysis
Source: Evid Based Complement Alternat Med. 2018 Sep 4;2018:7362340. doi: 10.1155/2018/7362340 (PMC6142757; doi:10.1155/2018/7362340)
Supplement: Supplementary Materials — Supplementary File: forest plots of KPS, side-effects, and tumor-related pain (DOC). Supplement 1: forest plots of response rate, overall survival, and disease control rate (DOC). Supplement 2: sensitivity analysis (DOC). Supplement 3: publication bias of the meta-analysis (DOC). [file 7362340.f1.zip › Supplementary Materials/supplement1.doc]

**Fig1.** **Forrest plot of RR (Risk Ratio) for evaluation of response rate in fixed effect model.** The RR of chemotherapy response rate in Cinobufacini injection and chemotherapy group was compared with the chemotherapy group. Individual study is shown in the square with blue color, and the pooled datasets were shown in the diamond, representing the 95% conﬁdence interval (CI) of each study. RR > 1 implied a better chemotherapy response rate of the experimental group. The size of each investigation represented the weighting factor (1/SE) assigned to the study.

**Fig2. Forest plot of RR for evaluation of response rate in random effect model.** The RR of chemotherapy response rate in Cinobufacini injection and chemotherapy group was compared with the chemotherapy group. Individual study is shown in the square with blue color, and the pooled datasets were shown in the diamond, representing the 95% conﬁdence interval (CI) of each study. RR > 1 implied a better chemotherapy response rate of the experimental group. The size of each investigation represented the weighting factor (1/SE) assigned to the study.

**Fig3. Forest plot of RR for evaluation of overall survival in fixed effect model.** The RR of overall survival in Cinobufacini injection and chemotherapy group was compared with the chemotherapy group. Individual study is shown in the square with blue color, and the pooled datasets were shown in the diamond, representing the 95% conﬁdence interval (CI) of each study. RR > 1 implied a better overall survival of the experimental group. The size of each investigation represented the weighting factor (1/SE) assigned to the study.

**Fig4. Forest plot of RR for evaluation of disease control rate in random effect model.** The RR of disease control rate in Cinobufacini injection and chemotherapy group was compared with the chemotherapy group. Individual study is shown in the square with blue color, and the pooled datasets were shown in the diamond, representing the 95% conﬁdence interval (CI) of each study. RR > 1 implied a better disease control rate of the experimental group. The size of each investigation represented the weighting factor (1/SE) assigned to the study.

**Fig5. Forest plot of HR (hazard ratio) for evaluation of disease control rate in fixed effect model.** The HR of disease control rate in Cinobufacini injection and chemotherapy group was compared with the chemotherapy group. Individual study is shown in the square with blue color, and the pooled datasets were shown in the diamond, representing the 95% conﬁdence interval (CI) of each study. HR < 1 implied a better disease control rate of the experimental group. The size of each investigation represented the weighting factor (1/SE) assigned to the study.

**Fig6. Forest plot of HR for evaluation of disease control rate in random effect model.** The HR of disease control rate in Cinobufacini injection and chemotherapy group was compared with the chemotherapy group. Individual study is shown in the square with blue color, and the pooled datasets were shown in the diamond, representing the 95% conﬁdence interval (CI) of each study. HR < 1 implied a better disease control rate of the experimental group. The size of each investigation represented the weighting factor (1/SE) assigned to the study
