# Supplementary material for: Cinobufacini Injection Improves the Efficacy of Chemotherapy on Advanced Stage Gastric Cancer: A Systemic Review and Meta-Analysis
Source: Evid Based Complement Alternat Med. 2018 Sep 4;2018:7362340. doi: 10.1155/2018/7362340 (PMC6142757; doi:10.1155/2018/7362340)
Supplement: Supplementary Materials — Supplementary File: forest plots of KPS, side-effects, and tumor-related pain (DOC). Supplement 1: forest plots of response rate, overall survival, and disease control rate (DOC). Supplement 2: sensitivity analysis (DOC). Supplement 3: publication bias of the meta-analysis (DOC). [file 7362340.f1.zip › Supplementary Materials/supplement2 .doc]

**Fig1. Forest plot of RR for evaluation of KPS in fix and random effect models.** The RR of KPS in Cinobufacini injection and chemotherapy group was compared with the chemotherapy group. Individual study is shown in the square with blue color, and the pooled datasets were shown in the diamond, representing the 95% conﬁdence interval (CI) of each study. RR > 1 implied a better KPS of the experimental group. The size of each investigation represented the weighting factor (1/SE) assigned to the study.

**Fig2. Forest plot of RR for evaluation of leukocytopenia in fix and random effect models.** The RR of leukocytopenia in Cinobufacini injection and chemotherapy group was compared with the chemotherapy group. Individual study is shown in the square with blue color, and the pooled datasets were shown in the diamond, representing the 95% conﬁdence interval (CI) of each study. RR< 1 implied a reduced incidence of leukocytopenia of the experimental group. The size of each investigation represented the weighting factor (1/SE) assigned to the study.

**Fig3. Forest plot of RR for evaluation of grade III-IV leukocytopenia in fix and random effect models.** The RR of grade III-IV leukocytopenia in Cinobufacini injection and chemotherapy group was compared with the chemotherapy group. Individual study is shown in the square with blue color, and the pooled datasets were shown in the diamond, representing the 95% conﬁdence interval (CI) of each study. RR< 1 implied a reduced incidence of grade III-IV leukocytopenia of the experimental group. The size of each investigation represented the weighting factor (1/SE) assigned to the study.

**Fig4. Forest plot of RR for evaluation of nausea and vomiting in fix and random effect models.** The RR of nausea and vomiting in Cinobufacini injection and chemotherapy group was compared with the chemotherapy group. Individual study is shown in the square with blue color, and the pooled datasets were shown in the diamond, representing the 95% conﬁdence interval (CI) of each study. RR < 1 implied a reduced incidence of nausea and vomiting of the experimental group. The size of each investigation represented the weighting factor (1/SE) assigned to the study.

**Fig5. Forest plot of RR for evaluation of grade III-IV of nausea and vomiting in fix and random effect models.** The RR of grade III-IV of nausea and vomiting in Cinobufacini injection and chemotherapy group was compared with the chemotherapy group. Individual study is shown in the square with blue color, and the pooled datasets were shown in the diamond, representing the 95% conﬁdence interval (CI) of each study. RR < 1 implied a reduced incidence of grade III-IV of nausea and vomiting of the experimental group. The size of each investigation represented the weighting factor (1/SE) assigned to the study.

**Fig6. Forest plot of RR for evaluation of HFS in fix and random effect models.** The RR of HFS in Cinobufacini injection and chemotherapy group was compared with the chemotherapy group. Individual study is shown in the square with blue color, and the pooled datasets were shown in the diamond, representing the 95% conﬁdence interval (CI) of each study. RR < 1 implied a reduced incidence of HFS of the experimental group. The size of each investigation represented the weighting factor (1/SE) assigned to the study.

**Fig7. Forest plot of RR for evaluation of pain relieving in fix and random effect models.** The RR of pain relieving in Cinobufacini injection and chemotherapy group was compared with the chemotherapy group. Individual study is shown in the square with blue color, and the pooled datasets were shown in the diamond, representing the 95% conﬁdence interval (CI) of each study. RR > 1 implied a reduced incidence of cancer pain of the experimental group. The size of each investigation represented the weighting factor (1/SE) assigned to the study.

**Fig8. Forest plot of RR for evaluation of anemia in fix and random effect models.** The RR of anemia in Cinobufacini injection and chemotherapy group was compared with the chemotherapy group. Individual study is shown in the square with blue color, and the pooled datasets were shown in the diamond, representing the 95% conﬁdence interval (CI) of each study. RR < 1 implied a reduced incidence of anemia of the experimental group. The size of each investigation represented the weighting factor (1/SE) assigned to the study.

**Fig9. Forest plot of RR for evaluation of diarrhea in fix and random effect models.** The RR of diarrhea in Cinobufacini injection and chemotherapy group was compared with the chemotherapy group. Individual study is shown in the square with blue color, and the pooled datasets were shown in the diamond, representing the 95% conﬁdence interval (CI) of each study. RR < 1 implied a reduced incidence of diarrhea of the experimental group. The size of each investigation represented the weighting factor (1/SE) assigned to the study.

**Fig10. Forest plot of RR for evaluation of peripheral neuropathy in fix and random effect models.** The RR of peripheral neuropathy in Cinobufacini injection and chemotherapy group was compared with the chemotherapy group. Individual study is shown in the square with blue color, and the pooled datasets were shown in the diamond, representing the 95% conﬁdence interval (CI) of each study. RR < 1 implied a reduced incidence of peripheral neuropathy of the experimental group. The size of each investigation represented the weighting factor (1/SE) assigned to the study.

**Fig11. Forest plot of RR for evaluation of oral mucositis in fix and random effect models.** The RR of oral mucositis in Cinobufacini injection and chemotherapy group was compared with the chemotherapy group. Individual study is shown in the square with blue color, and the pooled datasets were shown in the diamond, representing the 95% conﬁdence interval (CI) of each study. RR < 1 implied a reduced incidence of oral mucositis of the experimental group. The size of each investigation represented the weighting factor (1/SE) assigned to the study.
