# Supplementary material for: Cinobufacini Injection Improves the Efficacy of Chemotherapy on Advanced Stage Gastric Cancer: A Systemic Review and Meta-Analysis
Source: Evid Based Complement Alternat Med. 2018 Sep 4;2018:7362340. doi: 10.1155/2018/7362340 (PMC6142757; doi:10.1155/2018/7362340)
Supplement: Supplementary Materials — Supplementary File: forest plots of KPS, side-effects, and tumor-related pain (DOC). Supplement 1: forest plots of response rate, overall survival, and disease control rate (DOC). Supplement 2: sensitivity analysis (DOC). Supplement 3: publication bias of the meta-analysis (DOC). [file 7362340.f1.zip › Supplementary Materials/supplement3.doc]

|  |  |
| --- | --- |
| response rate | disease control rate |
|  |  |
| KPS | leukocytopenia |
|  |  |
| nausea and vomiting | diarrhea |

**Fig1.Egger’s publication bias plots**
